# Evaluation of clinical comorbidities in cognitively impaired patients with depressive symptoms

**DOI:** 10.1590/S1980-57642008DN10400013

**Published:** 2007

**Authors:** Rossana Maria Russo Funari, Letícia Lessa Mansur, Paulo Rogério Rosmaninho Varandas, Maria Isabel D'Avila Freitas, Wilson Jacob Filho

**Affiliations:** 1 Serviço de Geriatria do Hospital das Clínicas da Faculdade de Medicina da Universidade de São Paulo.; 2 Departamento de Fisioterapia, Fonoaudiologia e Terapia Ocupacional. Faculdade de Medicina da USP.; 3 Pós-Graduação Departamento Neurologia - FMUSP.; 4 Departamento de Clínica Médica da Faculdade de Medicina da Universidade de São Paulo.

**Keywords:** dementia, depressive symptoms, comorbidities, depression, demência, sintomas depressivos, comorbidades, depression

## Abstract

In demented patients, depressive symptoms as well as cognition and clinical comorbidities can interact and induce a complex condition of severity and handicap.

**Objective:**

The objective of this study was to evaluate the influence of depressive symptoms in cognitively impaired patients on associated clinical comorbidities in geriatric patients.

**Methods:**

One-hundred-thirty-eight (138) patients were divided into two groups: the first contained cognitively impaired patients with depressive symptoms while the second comprised cognitively impaired patients without depressive symptoms. To quantify comorbidities, the Modified CIRS Scale was used.

**Results:**

Out of the 138 patients, 52 were cognitively impaired with depressive symptoms and 86 were cognitively impaired without depressive symptoms, both having mean CDR of 1.74 (moderate dementia). The patients with depressive symptoms used more drugs (4.98 per patient vs. 3.45 per patient without depressive symptoms; p=0.001), presented more comorbidities (3.24 per patient vs. 2.46 per patient without depressive symptoms; p=0.009). However, these comorbidities were neither more severe nor more complex in the patients with depressive symptoms, with mean Comorbidity Severity Index of 1.45 in patients with and 1.37 in patients without depressive symptoms (p= 0.078) and mean Comorbidity Complexity Index of 2.41 in patients with depressive symptoms and 2.01 in those without depressive symptoms (p=0.103).

**Conclusion:**

Cognitively impaired patients with depressive symptoms had a greater absolute number of comorbidities and took more drugs although these comorbid diseases were less severe and complex than in non-depressive cognitively impaired patients.

## Abstract (pt)

Em pacientes com demência, sintomas depressivos, cognição e comorbidades podem interagir resultando em uma condição de agravamento do quadro e desvantagem.

**Objetivo:**

O objetivo do estudo foi avaliar a influência de sintomas depressivos sobre as comorbidades em pacientes geriátricos com declínio cognitivo.

**Metodos:**

138 pacientes foram divididos em dois grupos: o primeiro composto de pacientes com distúrbio cognitivo associado a sintomas depressivos e o segundo de pacientes com distúrbio cognitivo sem sintomas depressivos. Para quantificar as comorbidades a Escala CIRS modificada foi utilizada.

**Resultados:**

52 pacientes apresentavam distúrbio cognitivo com sintomas depressivos e 86 apresentavam distúrbios cognitivos sem sintomas depressivos, ambos com média de 1.74 no CDR (demência moderada). Os pacientes com sintomas depressivos usaram maior número de fármacos (4.98 / paciente com sintomas depressivos versus 3.45 / pacientes sem sintomas depressivos; p=0.001), apresentaram maior número de comorbidades (3.24 / paciente com sintomas depressivos versus 2.46 / pacientes sem sintomas depressivos; p=0.009) mas essas comorbidades não foram mais graves nem mais complexas nos pacientes depressivos: média do Índice de Gravidade das Comorbidades=1.45 no grupo com sintomas depressivos e 1.37 no grupo sem sintomas depressivos (p=0.078) e média de do Índice de Complexidade da Comorbidade com sintomas depressivos = 2.41 no grupo com sintomas depressivos e 2.01 no grupo sem sintomas depressivos (p=0.103).

**Conclusão:**

Os pacientes com distúrbio cognitivo associado a sintomas depressivos apresentaram maior número absolute de comorbidades e ingeriam maior número de fármacos mas as doenças eram menos graves e complexas do que as do grupo de não depressivos.

Mood alterations can affect a considerable percentage of individuals with dementia.^1,2^ Approximately 30 to 40% of patients with Alzheimer’s disease present significant depressive symptoms^3^ and the prevalence of depression in vascular dementia can be even greater.^4,5^ Patients with depression usually present more complaints and can present greater functional compromise than those not depressed,^6^ although diagnosis of depression in patients with dementia remains a challenge.^7^ Numerous studies have demonstrated how comorbidities, frequently called psychosocial, such as agitation, aggression, pain and depression can reduce the quality of life in the patient as well as caregiver.^8,9^ To our knowledge, there have been no studies analyzing possible influences of depression on clinical comorbidities in ambulatory patients with dementia.

The objective of this study was to verify the impact of depression in patients with cognitive disturbances considering 1) the number, severity and complexity of comorbidities; 2) number of medications used. The study included a descriptive analysis of the two groups, regarding the most prevalent comorbidities and the type and degree of dementia.

## Methods

138 patients with cognitive disturbances were selected at the Cognitive Geriatric Outpatient Unit - HC/FMUSP from 2000 to 2007. Patients were divided into two groups: those having dementia with depressive symptoms (DEM+DEP) and those having dementia without depressive symptoms (DEM). A multidisciplinary team evaluated all patients. The criteria used to diagnose dementia were from the National Institute of Neurological and Communicative Disorders and Stroke and Alzheimer’s Disease and Related Disorders Association (NINCDS-ADRDA). This classifies probable or possible Alzheimer’s disease^10^ while the criteria of the National Institute of Neurological Disorders and Stroke and Association Internationale pour la Recherche et l’Enseignement en Neurosciences (NINDS/AIREN) classifies vascular dementia as probable or possible,^11^ and the Diagnostic and Statistic Manual for Mental Diseases, 4th edition (DSM-IV) assesses dementia and depressive symptoms.^12^ The Mini-Mental State Examination (MMSE),^13^ the Clinical Dementia Rating (CDR),^14^ the Geriatric Depression Scale (GDS 30)^15^ and the modified Cumulative Illness Rating Scale (CIRS)^16^ were also applied to evaluate the degree and complexity of comorbidities.

These patients were submitted to clinical and laboratory exams, diagnosing current and previous diseases, and were questioned about the number of medications currently being taken.

Pearson’s chi-square test or Fisher exact test were used to compare categorical variables. Student’s t test or Mann-Whitney test were used for quantitative variables and either Pearson or Speaman coefficients were used for correlations between variables according to the presence or absence of normal distribution of the data.^17^ The value of statistical significance accepted was 0.05.

## Results

Of the 138 patients, 52 belonged to the DEM+DEP group and 86 the DEM group. The characteristics of each group such as age, gender, schooling and severity of dementia, are listed in Table 1. The proportion of male individuals was higher in the DEM group. The two groups presented average MMSE and CDR of 15.9 and 1.7 respectively (moderate dementia).

**Table 1:** Characteristics of the two groups studied regarding age, gender, schooling and severity of dementia.

	Age (years) mean (SD)	Gender Male/Female	Schooling (years) mean (SD)	CDR mean (SD)	Mmse mean (SD)
DEM+DEP	77.3 (6.95)	8/44	4.66 (5,01)	1.74 (0.87)	15.89 (7,42)
DEM	77.2 (6.69)	37/49	4.69 (3.97)	1.73 (0.88)	15.97 (8.64)

DEM: dementia; DEM+DEP: dementia with depressive symptoms; CDR: clinical dementia rating scale; MMSE: mini-mental state examination; SD: standard deviation.

**Figure 1:**
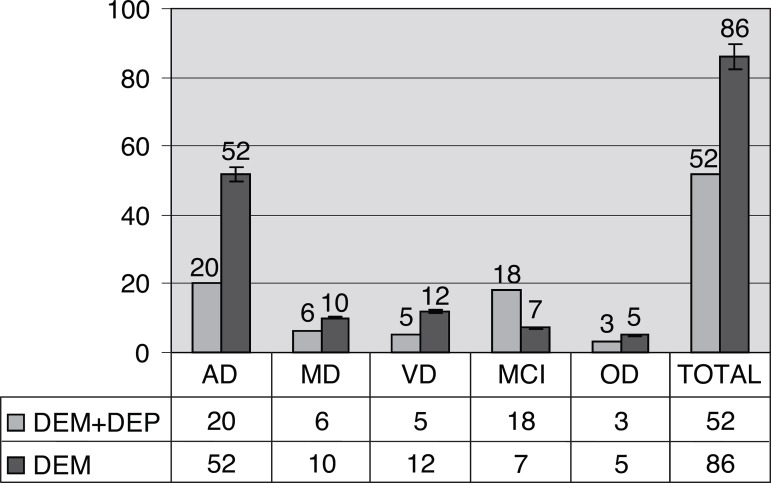
Diagnoses of dementias in patients with dementia and depressive symptoms, and dementia without depressive symptoms. AD: Alzheimer disease; MD: mixed dementia; VD: vascular dementia; MCI: mild cognitive impairment; OD: other dementias; DEM+DEP: dementia with depressive symptoms; DEM: dementia without depressive symptoms .

**Figure 2:**
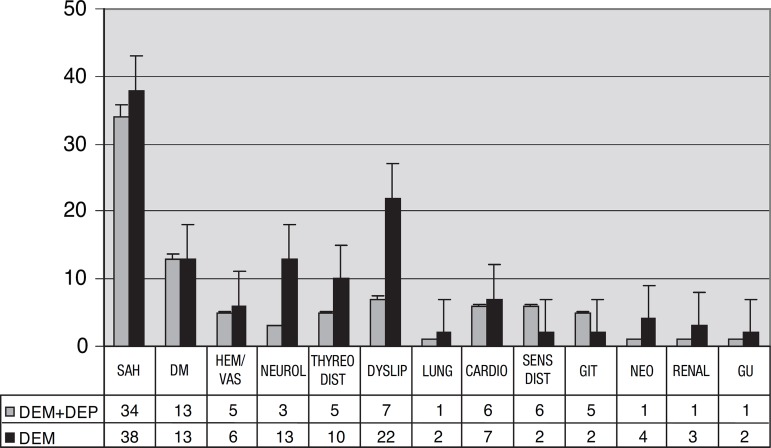
Most prevalent comorbidities in patients with dementia and depressive symptoms, and dementia without depressive symptoms. SAH, systemic arterial hypertension; DM, diabetes mellitus; HEM/VAS, hematopoietic and vascular diseasea; THYREO, diseases of thyroid; DYSLIP, dyslipidemias; LUNG, lung diseases; CARDIO, cardiopathies; SENDIST, sensorial disturbances; GIT, diseases of gastrointestinal tract; NEO, neoplasias; RENAL, renal diseases; GU, genito-urinary diseases.

The diagnoses for types of dementia along with the most prevalent comorbidities are listed in Figures 1 and 2.

The most prevalent comorbidities were arterial hypertension, mellitus diabetes and dyslipidemia in both groups, with neurological disorders and dyslipidemia being more prevalent in the group without depressive symptoms, and sensorial disturbances more present in the group with depressive symptoms.

**Figure 3:**
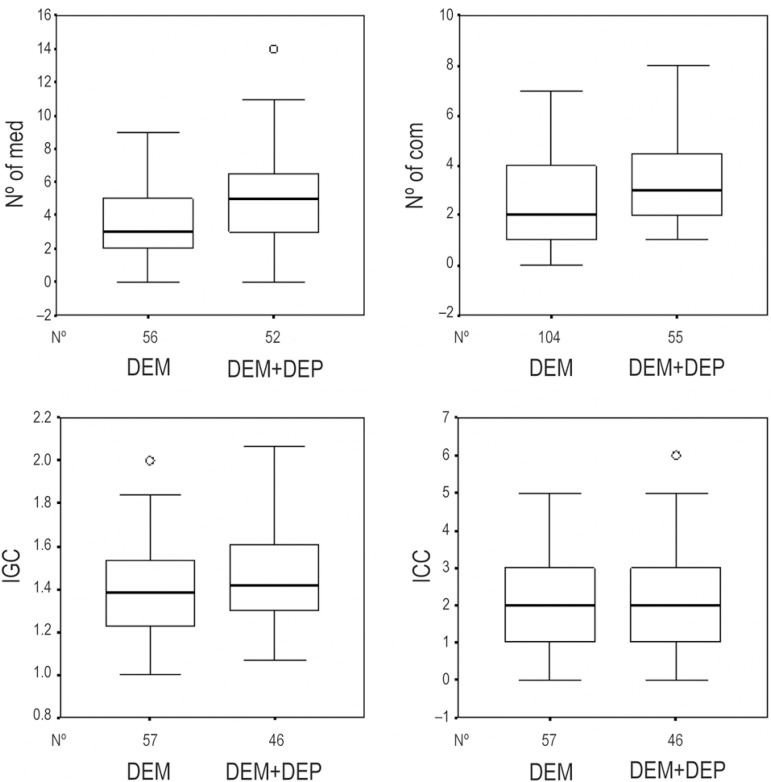
Box plots of the Number of medications, Number of comorbidities, comorbidity severity index, comorbidity complexity index for depressive symptoms. IGC: comorbidity severity index; ICC: comorbidity complexity index; DEM: dementia without depressive symptoms; DEM+DEP: dementia with depressive symptoms.

The patients with dementia and depressive symptoms took more medication: 4.98 per patient versus 3.45 medication per patient without depressive symptoms (p=0.001) and presented a greater absolute number of comorbidities 3.24 versus 2.46 per patient (p=0.009), however these comorbidities were neither more severe nor more complex, according to the CIRS scales: average CSI (comorbidity severity index) with depressive symptoms was 1.45 and without depressive symptoms 1.37 (p=0.078) and average CCI (comorbidity complexity index) with depressive symptoms was 2.41 and without depressive symptoms 2.01 per patient (p=0.103) (Tables 2 and 3 and Figure 3).

**Table 2:** Averages summary of the number of medications, number of comorbidities, comorbidity severity index, comorbidity complexity index for Dementia without depressive symptoms, and Dementia with depressive symptoms.

Averages summary	NM	NC	CSI	CCI
**DEM**				
N Mean Median Minimum Maximum Standard deviation	86 3.45 3.00 0.00 9.00 2.23	104 2.46 2.00 0.00 7.00 1.75	87 1.37 1.38 1.00 2.00 0.22	87 2.01 2.01 0.00 5.00 1.30
**DEM+DEP**				
N Mean Median Minimum Maximum Standard deviation	52 4.98 5.00 0.00 14.00 3.05	55 3.24 3.00 1.00 8.00 1.79	46 1.45 1.42 1.07 2.07 0.25	46 2.41 2.00 0.00 6.00 1.42

NM: number of medications; NC: number of comorbidities; CSI: comorbidity severity index; CCI: comorbidity complexity index; DEM: dementia without depressive symptoms; DEM+DEP: dementia with depressive symptoms.

**Table 3:** Inferential comparative results of average values for number of medications, number of comorbidities, comorbidity severity index, comorbidity complexity index, for dementia without depressive symptoms, and dementia with depressive symptoms.

Variable	Conclusion
Number of medications	DEM<DEM+DEP (p=0.001)
Number of comorbidities	DEM<DEM+DEP (p=0.009)
Comorbidity severity index	DEM=DEM+DEP (p=0.078)
Comorbidity complexity index	DEM=DEM+DEP (p=0.103)

DEM: dementia without depressive symptoms; DEM+DEP: dementia with depressive symptoms.

## Discussion

The findings of this study showed that when comparing two groups with very similar clinical characteristics, the group of patients with dementia associated to depressive symptoms presented a greater number of comorbidities and took a greater number of medications than non-depressive patients with dementia, although these comorbidities were neither more severe nor more complex. The majority of these illnesses did not interfere with basic activities, did not need treatment, and according to the morbidity scale used had excellent prognosis. Schubert et al., compared the profile of patients in a primary evaluation and concluded that multiple morbidities are common in the aged with or without dementia having a mean chronic disease frequency of 2.4 per patient and an average of 5.1 for medications.^18^ Kaup et al., compared dementia patients with depressive symptoms and depressive patients without dementia, and found a significantly positive association with greater number of comorbidities without dementia and with depressive symptoms, as well as greater physical dependency, independent of cognitive state.^19^ Thus, thorough diagnosis is necessary in dementia patients with depressive symptoms for correct treatment,^20^ although it is also important to value complaints and quantify morbidities to avoid excessive use of medication, as was seen in this study.

The most frequent comorbidities in both groups were hypertension and diabetes, while in a multi-centric study with ambulatory patients (REAL FR) cardiovascular diseases (including systemic arterial hypertension) were most frequent in patients with Alzheimer’s dementia, followed by sensorial alterations and neurological disturbances.^21^

In the present study, the patients with depressive symptoms associated to dementia presented a greater prevalence of sensorial disturbances in relation to patients with dementia and no depressive symptoms. Perhaps these sensorial deficits (in this case visual and auditory) may contribute to the decrease in quality of life in these patients and consequently may have constituted an additional factor for the emergence of depressive symptoms in our sample.

In the literature, numerous studies have related depressive symptoms with cognitive disturbances and dementia, although it is not yet clear if the depressive symptoms contributed to the worsening of cognitive results.^22^ Other studies have commented that patients with depressive symptoms and dementia need more inpatient healthcare services and nursing homes, as the depressive symptoms associated with dementia can affect the course of the illness, elevating the degree of functional alteration and leading to the potential risk of being institutionalized or hospitalized.^23^

It would be interesting for a further study to verify whether these patients with depressive symptoms are being correctly treated with adequate doses of medications, as it is known that patients who are poorly diagnosed and treated with sub-doses have a poorer clinical evolution^24^ while these two groups of depressed patients without dementia could be compared and analyzed to ascertain the impact of clinical morbidities on the daily lives of these patients. Thus, this study concluded that individuals with dementia associated to depressive symptoms presented a greater absolute number of comorbidities, although these tended to be less severe and less complex than in the group without depressive symptoms. It is of the utmost importance to observe details on cognitive disturbances in association with depressive symptoms, thereby avoiding poly-pharmacy, found to be the most prevalent phenomenon in the group of patients with depressive symptoms and dementia.
